# Toward Fullerene-Free PIN Perovskite Solar Cells

**DOI:** 10.1021/acsenergylett.5c02987

**Published:** 2025-11-18

**Authors:** Kelly Schutt, Melissa Davis, Muzhi Li, Samuel A. Johnson, Daniel Martinez, Jochen Titus, Tomas Leijtens, Blake Martin, Michael D. McGehee, Seth R. Marder, Nicholas Rolston, Joseph M. Luther

**Affiliations:** † 53405 National Renewable Energy Laboratory, Golden, Colorado 80401, United States; ‡ Materials Science and Engineering, Fulton Schools of Engineering, 7864 Arizona State University, Tempe, Arizona 85287, United States; § Renewable and Sustainable Energy Institute, 1877 University of Colorado, Boulder, Colorado 80309, United States; ∥ Swift Solar, 981 Bing Street, San Carlos California 94070, United States; ⊥ Sofab Inks, 11351 Decimal Drive, Louisville, Kentucky 40299, United States

## Abstract

We highlight opportunities for a transformative shift in perovskite solar cell design by expanding electron transport layers (ETLs) beyond fullerenes. Fullerenes have known limitations, including constraints on open-circuit voltage, stability, and mechanical integrity. Recently, fullerene-free p-i-n cells with power conversion efficiencies exceeding 25% have been demonstrated via both naphthalene diimide-SnO_*x*_ bilayers and nonfullerene acceptor-based ETLs. Despite successes, fullerenes remain the de facto ETLs for perovskites. Drawing lessons from organic photovoltaics, where it took decades to transition from fullerenes to more broadly available and efficient materials, we explore pathways to accelerate the development and adoption of fullerene-free ETLs. This requires understanding the similarities and differences between organic and perovskite solar cells, which will necessitate carefully designing fullerene replacements with both, high efficiency and also, critically, durability under operation. Here, we incorporate literature data to facilitate comparisons, and independently conduct fracture energy measurements for alternative ETL configurations to motivate their adoption.

## Graphical abstract

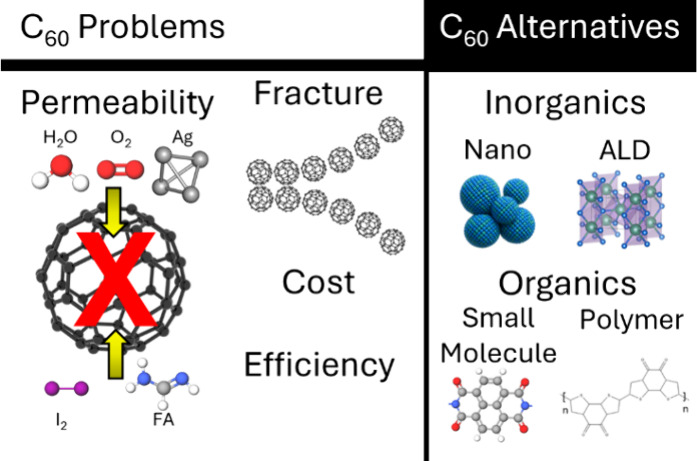

Advances in perovskite composition, device architecture, contacts, passivation, and packaging have driven the efficiency and stability of perovskite solar cells to levels that are closer to the stringent demands of commercially viable products with multidecadal lifetimes. In contrast to this rapid progress over the past decade, there is a persistent and limiting reliance on fullerenes as electron extraction layers. In this work, we combine an analysis of past results with original mechanical data to provide perspective on strategies for fullerene-free devices.

Fullerene layers in contact with perovskites represent the largest source of voltage loss in perovskite device stacks, since the wave function overlap of the perovskite and fullerene induces trap states at the interface. Fullerenes are also a key limiting factor for long-term stability.^1–6^ The surface recombination velocity for the perovskite–C_60_ interface is ∼2 × 10^3^ cm s^–1^, while for perovskite on the self-assembled monolayer HTL, 2PACz, it is as low as 12 cm s^–1^, making passivation at the ETL interface crucial for high performance.^7–9^ With respect to durability, fullerenes are permeable to the egress of volatile species that result in irreversible degradation to the perovskite, fullerenes permit oxygen and moisture ingress, and fullerenes are vulnerable to metal contact diffusion. Most importantly, fullerene–perovskite interfaces typically have the lowest fracture energy (*G*_c_) of all interfaces present in photovoltaic devices and lead to catastrophic failure in fielded modules, as we discuss later.^4,10^ While less of a concern than their durability and efficiency limitations, fullerenes are also expensive. Technoeconomic analyses find that C_60_ is one of the costliest materials in a perovskite module, exceeded only by the transparent conductive oxide, glass, encapsulants, and, if used, the SnOx precursor of (tetrakis)­dimethylamido tin­(IV). At lab scale, sublimed grade C_60_ costs ∼$100 g^–1^, while 1000 kg scale pricing is ∼$18 g^–1^.^11,12^ Low throughput sublimation purification is required to avoid progressive electronic degradation of C_60_ during continuous evaporation processes,^13^ which compounds the cost disadvantage and adds to the embodied energy of fullerenes. The embodied energy of fullerenes, ∼65–107 GJ/kg, is also an order-of magnitude higher than some organic molecules, which sets a floor on cost reductions through economies of scale.^14,15^

Despite these drawbacks, fullerenes are still ubiquitous in perovskite solar cell research, and the highest efficiency p-i-n cells have thus far relied on them for electron transport layers (ETLs). Several factors contribute to the continued use of fullerene, including familiarity, commercial availability, and the absence of extensive efforts to develop alternative materials. In many cases, researchers have conducted tests of nonfullerene acceptors developed for high-performance organic solar cells without redesigning the molecules to account for the specific differences in requirements of these organic versus perovskite solar cells. Perovskite and organic solar cells have distinct requirements, such as PSC’s need for ETL transparency and resistance to reactive halides and mobile ion species, which are not factors in organic solar cells. Notably, the drawbacks of fullerenes for organic solar cells were known, yet it took over 20 years to replace fullerenes with materials that were widely adopted and produced more efficient cells.^16,17^ Fullerenes offer several benefits: wide commercial availability with batch-to-batch consistency, reasonable LUMO alignment with perovskites where *E*_g_ < ∼1.6 eV, sufficient charge-carrier mobility, high film uniformity when solution and vapor deposited, and an isotropic electronic surface that makes molecular orientation irrelevant to device-level performance, thereby broadening the deposition process window.

The search for fullerene replacements is well underway with several candidate materials being explored; these materials can be broadly grouped into organics, inorganics, and organic–inorganic hybrids as shown in Figure 1A. Organics include small molecules, such as naphthalene and perylene diimides (NDI and PDI), and the donor–acceptor–donor motifs in fused ring electron acceptors (FREAs) such as Y6.^18,19^ Inorganic ETLs include metal oxides deposited from nanoparticle solutions or thin films grown via atomic layer deposition (ALD), although combination approaches of nanoparticles with subsequent ALD in-filling have also been used.^20–22^ Hybrid approaches typically isolate the metal oxide with a thin organic interlayer, which serves as a protective barrier for ALD growth processes that would otherwise react with the perovskite surface and inhibit charge extraction.^23–26^ We compare these fullerene alternatives in terms of their reproducibility, reported efficiency, fracture energy, permeability (water vapor transmission rate), mobility, transmittance, and cost (Figure 1B). The plot is designed so that the outermost region is the most desirable. C_60_ stands out only for offering greater reproducibility and efficiency than the alternatives. Moreover, although the metrics summarized in Figure 1B capture the general factors determining whether an ETL is suitable for the successful commercialization of high-efficiency PSCs, additional mechanisms specific to perovskite systems—yet not fully understood or systematically investigated—must also be considered. These aspects, while challenging to address, are essential for improving device reliability. For instance, ion migration at the perovskite/nonfullerene ETL interface may critically influence device stability, as interfacial reactivity can generate byproducts detrimental to long-term stability.

**Figure 1:**
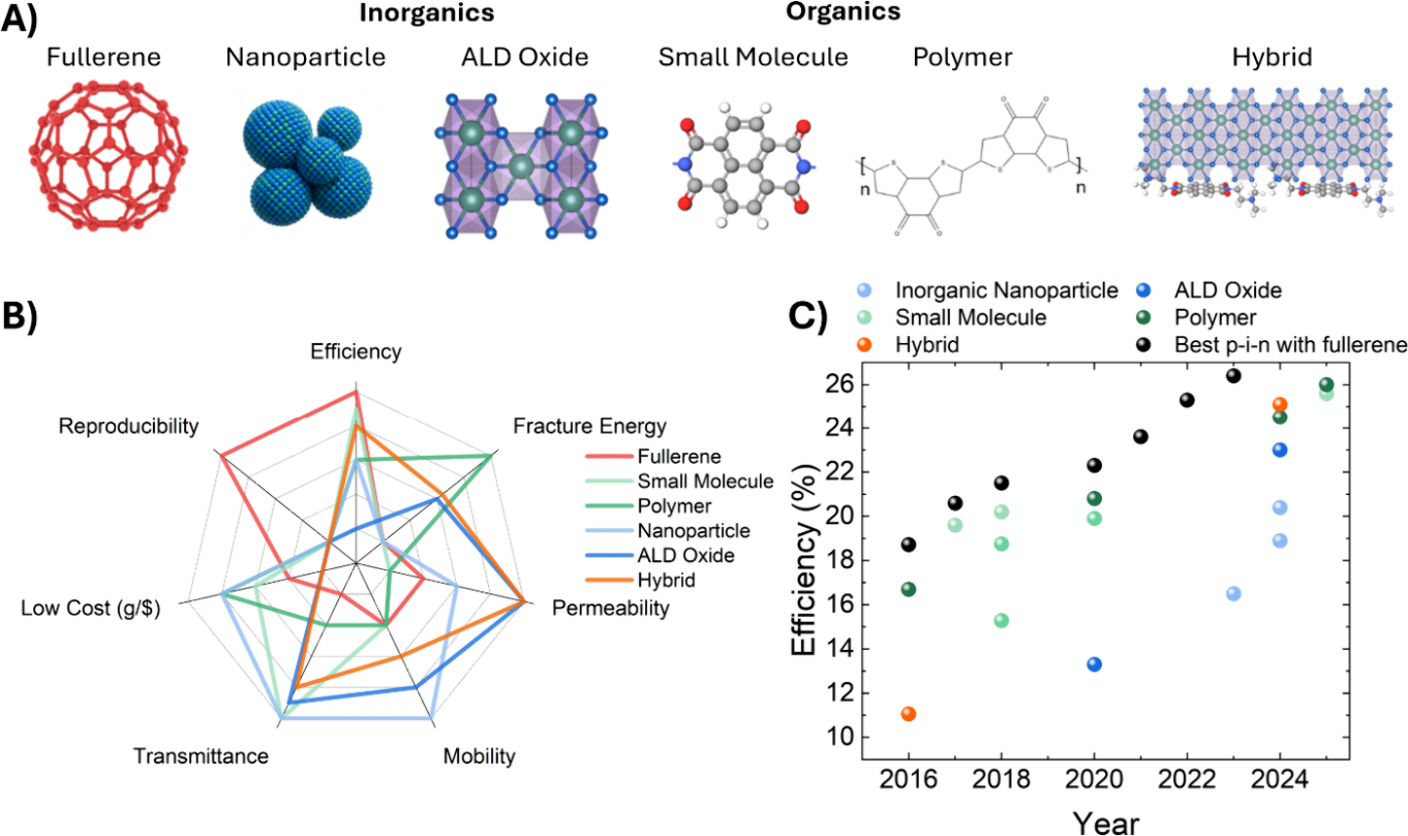
Properties of electron transport layers. (A) Diagram of classes of materials explored as alternative ETLs. Oxide layers can be formed via nanoparticle routes or ALD. Small molecule and polymer examples include naphthalene diimides and fused ring electron acceptors. Hybrid layers include an organic and an inorganic bilayer or blended ETL, such as a naphthalene diimide buffer layer that enables ALD SnO_*x*_ growth. (B) Spider plot comparison of desired features of ETLs compared across the different classes of materials. The outermost edge of the plot is the most desirable region for device performance, reproducibility, and cost. To address inherent ambiguity in this high-level analysis, such as the absorption coefficient of small molecules (which varies and can exceed that of fullerenes), we provide quantitative values from prior literature used to generate the plot in Table S1, opting for device-relevant materials and thicknesses whenever possible. Search queries for the reproducibility metric are in Table S2. (C) Efficiencies of fullerene-free and fullerene-containing p-i-n cells over time. Each data point is sourced from previous literature, with references in Tables S2 and S3.

Alternatively, rather than complete replacement, there have also been many approaches to ameliorate the weaknesses of fullerenes, and to use fullerenes in tandem with other ETL interlayers.^27–33^ We briefly describe fullerene modifications since these directly address fullerene-related mechanical integrity, which is arguably the most pressing limitation for device durability. Recently, You et al. modified C_60_, adding a CH_2_–NH_3_^+^ cation and a Cl^–^ anion. The modified C_60_ devices exhibited a ∼3-fold greater *G*_c_ (∼1.4 J·m^–2^) relative to devices with unmodified C_60_. Mini-modules with the modified C_60_ retained 95% of their initial efficiency in an accelerated test (85 °C, 1500 h), while the C_60_ mini-modules retained just 62% of their initial efficiency.^30^ Polymeric additives and modifications to fullerene ETLs have also improved device stability and initial efficiency, prevented fullerene aggregates (up to a few microns across) from forming during thermal stressing, and enhanced the intermolecular interaction energy of the ETL by about 6-fold relative to C_60_.^31,33^ Ozone treatment of fullerenes prior to ALD SnO_*x*_ deposition also enhances interfacial adhesion by providing more favorable binding sites for Sn precursors during initial deposition cycles.^34^ Perovskite surface passivation with 5-ammonium valeric acid iodide can also enable ALD growth of Al_2_O_3_ without interfacial reactions, potentially pointing the way to thin interfacial layers that could also enable the growth of ALD SnO_*x*_.^35^ Carboranes, carbon–boron clusters with excellent thermal and oxidative stability and efficient carrier transport, have also been used to reduce trapping at perovskite–fullerene interfaces.^36,37^ Carboranes provide wide tunability through functional group additions and incorporation into polymers^38^ and could have use as stand-alone ETLs.

While these modifications alleviate some of the concerns with mechanical integrity, other limitations of fullerenes remain, including poor band alignment with wide gap perovskites, parasitic absorption in tandems, permeability, and cost. Therefore, we turn our focus on p-i-n devices without any fullerenes, highlighting both the strength and weakness of the various approaches. We restrict our scope to p-i-n cells since, relative to n-i-p counterparts, p-i-n cells exhibit improved durability and also enable transparent conductive oxide top contacts, making them preferred for use in tandems.^39,40^

Since 2018, several groups have reported fullerene-free p-i-n cells with organic replacements having power conversion efficiencies above 20% (Figure 1C); however, these strategies have not achieved broad adoption, and in many cases, there have not been independent reports reproducing the initial observation.^18–20,18–20,41–54^ Before exploring the classes of fullerene alternatives in detail, we offer a few considerations that could lead to greater adoption of the new fullerene alternatives:

1. Power conversion efficiencies that have been certified will improve confidence in the architecture, even when the efficiency is not record-breaking. Most of the results in Figure 1C are not certified, and a few of the published IV curves do not show both forward and reverse scans or stabilized power output, making an accurate power conversion efficiency difficult to assess. If independent certification is not feasible due to budget or other restrictions, then the forward and reverse scans and the stabilized power output should be reported, along with the IV scan parameters (including step size, sweep rate, prebiasing, light soaking, etc.).
2. Stability testing implementing ISOS testing protocols for typical operating conditions under stress (e.g., at 85 °C and 1 sun illumination) and a control device with a fullerene ETL that is directly compared as a stability benchmark.^55^
3. Demonstration of lab-to-lab reproducibility based upon the methods sections that provide sufficient detail required to ensure process transferability.

## Fullerene Mechanical Failures

While the perovskite community continues to advance the efficiency of PSCs, increasing attention is being directed toward enhancing their stability. In particular, the mechanical integrity of PSCs has been identified as a significant hurdle that must be addressed for successful commercial adoption of this PV technology. A major contributor to the fragility of perovskite devices is the use of fullerene-based materials as ETLs within the device stack.^4,56^ C_60_ and its derivatives like phenyl-C61-butyric acid methyl ester (PCBM) have been reported to exhibit weak bonding both internally and at interfaces with adjacent layers in PSCs, primarily due to the limited intermolecular interactions governed by van der Waals forces. This means that they are susceptible to both interfacial delamination from poor adhesion to the underlying perovskite and overlying electrode, as well as internal cohesive fracture due to low intermolecular bonding energies­(∼0.44 eV).^57^ Fullerene-related delamination has been observed under an electron microscope in small area lab cells (Figure 2A) and at macroscopic scales in outdoor tests of perovskite modules (Figure 2B), making fullerene fragility a critical issue for scaling perovskite PV.

**Figure 2:**
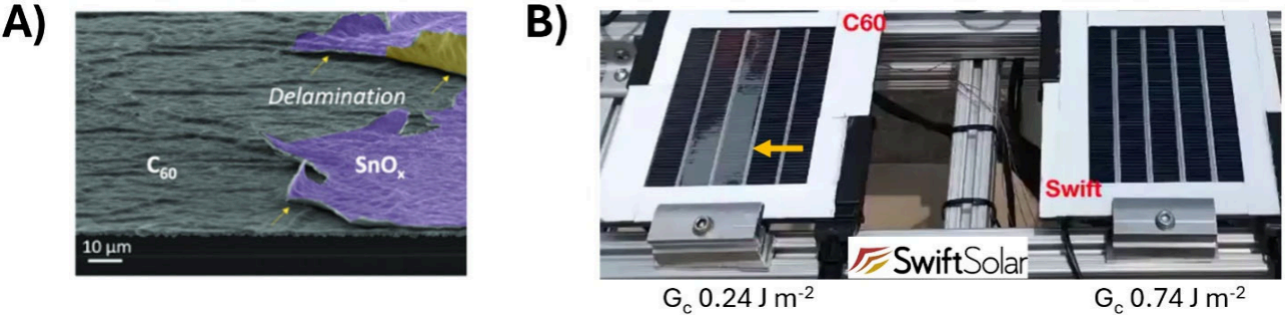
Fullerene-related delamination. (A) Scanning electron microscope image demonstrating delamination between C_60_ and SnO_*x*_. The purple area corresponds to Ag/MgF_2_ electrode while the yellow area represents the lift-off film that delaminated. Reproduced with permission from ref 56. Copyright 2022 ACS. (B) Photograph of modules under outdoor test at NREL with C_60_ and a proprietary, fullerene-free ETL. The gray portion of the C_60_-containing module indicated by the arrow exhibits severe delamination, which may be ascribed to the lower *G*_c_ of C_60_. This figure contains an original observation.

Quantifying mechanical integrity in multilayer structures is commonly achieved by measuring the *G*_c_ of a PSC, which is considered the most critical parameter for evaluating mechanical reliability.^58,59^ A high *G*_c_ will help limit crack propagation and delamination when the film is subject to stresses/strains that develop from processing and operation, driven by effects such as a coefficient of thermal expansion mismatch from the device layers and the underlying substrate, and from external mechanical stresses during fielded operation (such as wind shear, mechanical vibration, impacts from hail, etc.). In this work, we used a double cantilever beam test as a reproducible and accurate method for measuring *G*_c_ of PSCs and partial film stacks.

Figure 2B shows photos of outdoor stability testing conducted by Swift Solar, revealing severe delamination in devices at the C_60_ interface, while those employing a proprietary, nonfullerene based ETL, “ETL F”, retained their mechanical integrity. This delamination behavior correlates with measured *G*_c_, where C_60_-based devices exhibited a *G*_c_ of ∼0.2 J·m^–2^, compared to ∼0.7 J·m^–2^ for ETL F. Whether a *G*_c_ of ∼0.7 J·m^–2^ is sufficient for a 30-year lifetime is undetermined, but rapid thermal cycling could be used to investigate delamination-based failure across more ETLs. The International Electrotechnical Commission 61646 standard also requires 200 cycles from −40 to 85 °C. We realize that an anonymized “ETL F” is not useful for the community to adopt; however, confidentiality is required from materials produced in industry. We now provide our perspective on mechanically robust alternatives.

## Fullerene Alternatives: Energetics

Fullerene layers have appropriate electron affinities for charge extraction of low bandgap compositions (<∼1.6 eV) of perovskites with a particular conduction band energy. However, relative to organic compounds, the ability to tune both the optical and electronic properties is more limited. Consequently, as different perovskite compositions are being adopted in tandem solar cells, *V*_oc_ losses from misalignment will increase as the perovskite bandgap increases (Figure 3A).^60,61^ Furthermore, the availability of various inorganic oxides, whose properties can be further tailored through surface modifiers, also enables more flexible optimization approaches. For example, SnO_*x*_ can also offer better energetic alignment than fullerenes in single junction cells, depending on its doping and processing. The cell efficiency will be determined by factors beyond band alignment, including the ETL conductivity, thickness, defect density, chemical reactivity, and parasitic absorption. However, the band alignment is a useful starting point because it places an upper bound on the quasi-Fermi level splitting.

**Figure 3:**
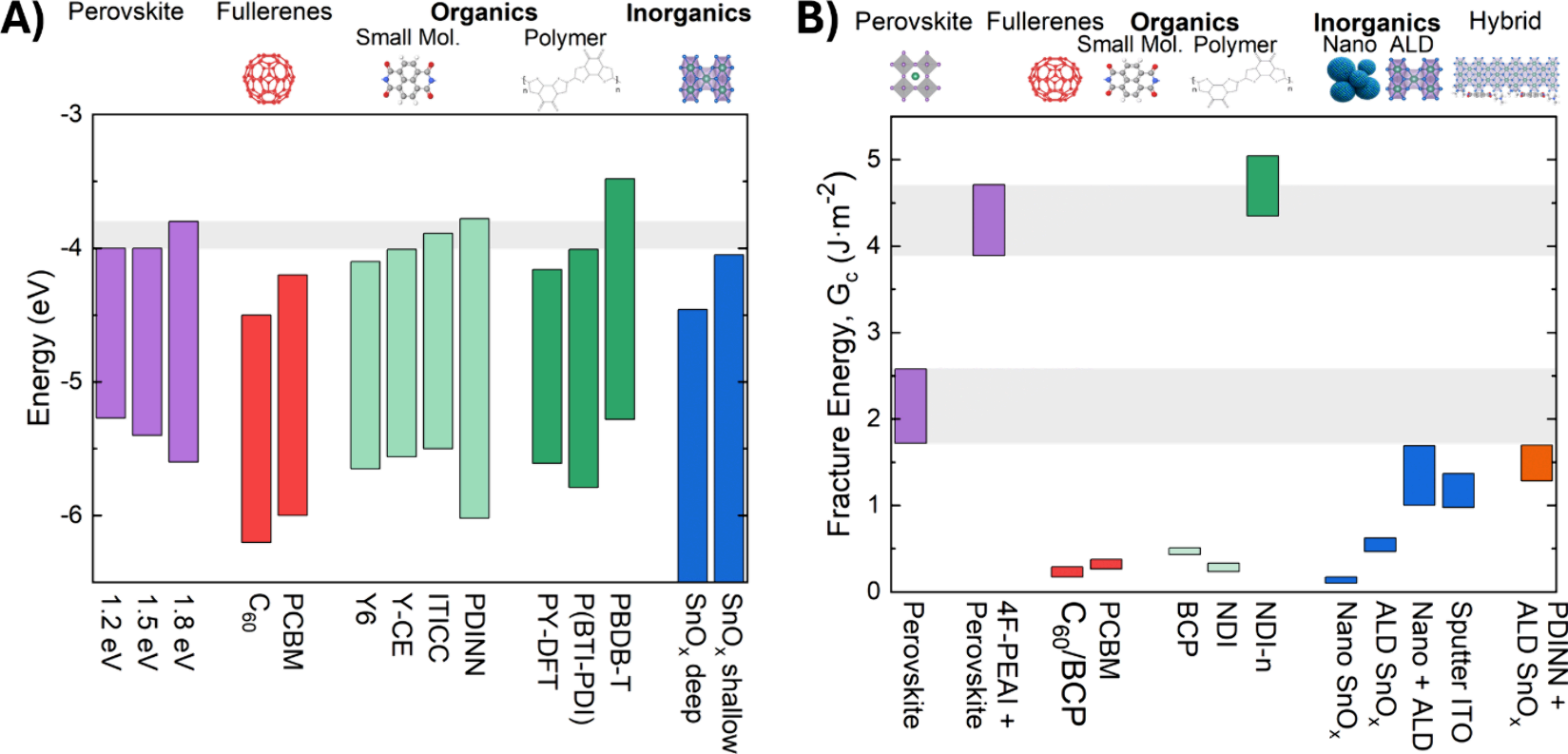
Band alignment and *G*_c_ of perovskites and electron transport layers. (A) The misalignment of the LUMO of fullerenes with perovskite conduction bands increases efficiency losses as the bandgap of the perovskite increases. The energy levels are shown for 1.2 eV (FA_0.7_MA_0.3_Pb_0.5_Sn_0.5_I_3_), 1.5 eV (FAPbI_3_), and 1.8 eV (CsPbI_2_Br) perovskites. Data for the energy levels are from previous literature, as detailed in Figure S4. (B) All materials evaluated had substantially lower *G*_c_ than bulk perovskite and passivated perovskite films, except for the polymeric NDI-n. “Nano + ALD” is SoFab nanoparticle SnO_*x*_ followed by growth of 15 nm of ALD SnO_*x*_. Sample preparation is described in Methods. This figure contains original data.

The perovskite band alignment with transport layers also strongly influences *J*_SC_ and device stability, since energy offsets drive the accumulation of halide ions at interfaces. This ion-induced field screening is a dominant factor in intrinsic device stability because it reduces charge extraction even in the absence of chemical or mechanical vulernabilities.^62^ In one study, as the energy offset between the perovskite and charge transport layers increased, the degradation rate of devices also increased.^63^ Higher ion density at contacts could contribute to accelerated ion egress from the perovskite, which causes unintentional doping of contact layers, electrode corrosion, and irreversible degradation to the active layer.^64–66^ Therefore, ensuring long-term durability requires charge selective contacts with well-matched energy levels and fracture energies matching or exceeding the perovskite.

## Fracture Energy (*G*_c_)

In contrast to the abundant literature values for the energy levels of perovskites and contact layers, fracture energy data were scarce. Therefore, we gathered original data for various ETLs across each material category, creating a common reference by depositing each of them on the same perovskite in a p-i-n configuration. We then tested their *G*_c_ with the double cantilever beam technique (described in the Methods). To obtain a reliable data set, at least three samples of each architecture were measured, giving rise to the range of values shown in Figure 3B. Fracture may occur within either the ETL, the perovskite, or at the interface. As a reference point, the fracture *G*_c_ of c-Si solar cells ranges from ∼10–200 J·m^–2^,^4^ and we have reported an experimental threshold of >1 J m^–2^ as a criterion for mechanical robustness.^67^

There are three striking features of our data set:

1. With one exception, none of the ETLs tested reached the lower bound of the perovskite *G*_c_, although some came close. Therefore, the perovskite is not the weak layer in these architectures, and device toughness can be improved by addressing the ETL bonding and/or ETL–perovskite interface.
2. Passivating the perovskite with 4-fluoro-phenethylammonium iodide (4F-PEAI) roughly doubled the perovskite *G*_c_, a previously unreported result. One prior study found that the addition of 5-aminovaleric acid also improved the *G*_c_ of perovskite films and attributed the effect to increased plasticity and crack deflection around grain boundaries. Polymer additives to the perovskite bulk can also enhance *G*_c_.^68,69^ We speculate that some of the stability enhancement seen from passivating agents could arise from their contribution to the mechanical properties of the perovskite film; future passivation research could also report mechanical properties to investigate this possibility.
3. The polymer ETL NDI-n was the only ETL with greater *G*_c_ than the perovskite. This material, poly­(*N*-(5-(5-norbornene-2-carbonyl)­oxy)­pentyl)-*N*′-*n*-hexyl-naphthalene-1,8:4,5-bis­(dicarboximide), has previously been used as an ETL in n-i-p cells.^70^ While the *G*_c_ of other polymeric fullerene replacements has yet to be measured, polymer *G*_c_ can be increased by orders of magnitude through the modification of chain lengths, entanglement, and cross-linking, providing greater mechanical tunability and durability than other ETLs.^71^ Polycarbonate, for instance, has a *G*_c_ 3 orders of magnitude greater than the perovskite (a few kJ·m^–2^).^72^ Motivated by mechanical integrity, there may be a promising polymeric ETL design space, where functional groups could also be included to provide surface passivation or anchor to the perovskite surface. To date, the highest PCE for a polymeric NDI ETL is about 20% (Figure 1C).

Our data also show that small molecules, nanoparticles, and fullerenes have similar *G*_c_ when deposited on perovskite. This is because these materials lack strong chemical bonds (low cohesion) and have weak interaction at the interface or in the bulk (low adhesion). Either low cohesion or adhesion is a direct consequence of mechanically weak layers susceptible to delamination, and results in *G*_c_ values <∼0.5 J·m^–2^. Therefore, we do not expect small molecules or nanoparticles to provide long-term durability, unless appropriate modifications are undertaken.

We modified a nanoparticle SnO_*x*_, provided by Sofab Inks, with the deposition of ALD SnO_*x*_. This greatly improved the *G*_c_, making it among the toughest nonpolymeric ETLs we evaluated. SoFab SnO_*x*_ is proposed to function as a barrier layer that mitigates damage to the perovskite layer during ALD-SnO_*x*_ deposition, while simultaneously serving as an interfacial layer that promotes improved bonding with the perovskite surface. We also tested a hybrid ETL comprised of a protective aliphatic amine-functionalized perylene-diimide (PDINN) buffer layer that enabled ALD SnO_*x*_ in devices with PCE of ∼25%.^26^ We found the *G*_c_ was similar to the SnO_*x*_ nanoparticles with subsequent ALD growth. ALD SnO_*x*_ is one of the most promising options for a combined ETL and encapsulant due to its low permeability and high optoelectronic quality. Combining ALD SnO_*x*_ with the polymeric NDI-n or other polymers will yield further insight. Such observations raise questions, such as whether the ALD layer would provide further enhancement to the best-in-class ETL toughness or reduce the toughness compared to the polymer alone.

The approaches tested here do not represent the full landscape of possibilities. Numerous cross-linking chemistries remain unexplored and may be engineered to simultaneously achieve mechanical reinforcement and high electronic quality. In addition, relative to fullerenes and small molecules, fully inorganic ETL alternatives offer a promising route toward improved mechanical durability and operational stability, particularly when coupled with tailored surface treatments or interface engineering strategies.

## Inorganic Fullerene Alternatives

Relative to organic ETLs, metal oxides offer mobility that can be orders-of-magnitude higher, have thermal stability that far exceeds that of perovskite organic cations, and cost below $1 g^–1^ at lab scale. Of the metal oxides, ZnO, TiO_*x*_, and SnO_*x*_ have been widely utilized in perovskite solar cells due to their favorable band alignment, high mobility, optical transparency, and low cost. Unfortunately, all the commonly employed metal oxides in PSC are catalytic. Chemical instabilities arising from acid–base reactions with the organic cation in ZnO,^73^ and under UV light in TiO_*x*_,^74^ make SnO_*x*_ the favored material. However, even SnO_*x*_ suffers from interfacial chemical reactions with perovskite, where it deprotonates organic cations and cleaves Pb–I bonds.^75^ Therefore, inert, buffering interlayers are desirable for device durability.

There are several routes to SnO_*x*_ deposition including sol–gel, chemical bath, sputtering, ALD, and electrodeposition. In the p-i-n configuration, damage to the underlying perovskite restricts these options, ruling out aqueous processes and preventing annealing above ∼150 °C. Considering these limitations, SnO_*x*_ nanoparticles have been deposited from orthogonal solvents such as ethanol to achieve device power conversion efficiency (PCE) of ∼14%. When doped with yttrium to modify the SnO_*x*_ work function, the PCE of Sofab SnO_*x*_ nanoparticles improved further to 20.4%.^76^ However, the low thermal budget for p-i-n devices with a nanoparticle ETL restricts their fill factor, as explained below.^54^

N-i-p devices with a perovskite grown on a metal oxide have shown superior fill factors relative to p-i-n devices, where the metal oxide is deposited on top of the perovskite. This is because n-i-p devices permit a high temperature anneal (∼200–500 °C) of the metal oxide before the perovskite is deposited. This anneal drives off ligands, promotes film density through sintering, and modulates the oxygen vacancy concentration, thereby enhancing the electronic quality of the oxide. As one example, high temperature annealing of solution-processed SnO_*x*_ increases conductivity by 2 orders of magnitude relative to annealing at <200 °C.^77,78^ One possible approach to overcome the limited thermal budget of p-i-n devices is to use a pulsed ultraviolet laser or intense pulsed light (IPL) to locally anneal the metal oxide nanoparticles while limiting thermal damage to the underlying perovskite. Perovskite thin films can be polished with femtosecond lasers to improve device efficiency, and nanoparticle ETLs may be amenable to similar processing without damaging the underlying perovskite.^79^ This approach has shown promise for IPL processed SnO_*x*_ on FTO in n-i-p devices, with fill factors exceeding 78%, a figure that has yet to be achieved in p-i-n with nanoparticle metal oxide counterparts.^80^ Another possible approach is incorporating polymeric additives to form polymer–metal-oxide nanocomposites—both in the bulk and at interfaces—that could relax thermal-budget constraints and other processing limitations encountered when depositing layers over perovskite absorbers. Additionally, this type of hybrid system could further tune energetic alignment with the perovskite and increase the mechanical durability of the interface.

In contrast to nanoparticle metal oxides, ALD of SnO_*x*_ offers dense, conformal films and has been widely used to grow SnO_*x*_ on fullerene interlayers to provide an encapsulating ETL that can also withstand sputter damage (e.g., from transparent electrodes such as ITO).^81^ However, ALD SnO_*x*_ on bare perovskite, grown from the commonly used precursors of tetrakis­(dimethylamino)­tin­(IV) (TDMASn) and H_2_O, results in devices with PCE < 1%.^23–26^ Since the perovskite photoluminescence is essentially unchanged after deposition of ALD SnOx, the low device performance is attributed to a surface reaction between TDMASn and the perovskite. Deprotonation of formamidinium creates a thick surface PbI_2_ layer that passivates the perovskite but restricts charge extraction. Other ALD metal oxides, including Al_2_O_3_ and TiO_2_ have been successfully grown directly on perovskite surfaces without damage, or even with benefits to device performance, raising the possibility that novel SnO_*x*_ precursor chemistry could enable direct growth of this metal oxide on MHP films.^1,2^ The discovery of a compatible precursor could also enable a wider process window for buffering interlayers that prevent direct contact between the perovskite and SnO_*x*_, where pinholes, low packing density, or other defects would not be catastrophic.

## Organic and Hybrid Fullerene Alternatives

Relative to metal oxides, organic fullerene alternatives offer a broader range of tunability through modifications to core, functional, and/or side chain groups that modify the LUMO level. They could passivate the perovskite interface, enhance solubility, and modify molecular packing for dense films with sufficient mobility. Recent demonstrations of fullerene-free perovskite cells with efficiencies above 25% have used fused-ring electron acceptors (FREAs), with the donor–acceptor–donor motif, and naphthalene diimides (NDIs).^26,51^

Both of these demonstrations used molecules that were adopted into perovskite architectures after first being used as materials for organic photovoltaics (OPV). We believe the perovskite community should instead design fullerene alternatives specifically for its needs, since some of the design criteria for OPV are irrelevant or may even be detrimental to perovskite devices, and because unnecessary synthetic complexity increases costs. OPVs are designed for a bulk heterojunction architecture necessitated by short exciton diffusion lengths, and their materials must also strongly absorb light. The bulk heterojunction makes material morphology and intermixing of paramount importance in OPV, which results in many OPV molecules having a twisted, nonplanar conformation (even if the core or backbone is planar) or packing in disordered networks.^82–85^ Planar molecules may be more suitable for perovskite PV; by offering a higher packing density they can also reduce permeability and improve mobility through a greater overlap of bonds and reduced carrier hopping distances. The short exciton diffusion length in OPV has also driven the donor–acceptor–donor motif to efficiently separate excitons, but this adds unnecessary complexity for perovskites. While OPV materials have also been designed to maximize light absorption, this only reduces current density in perovskites (for reflected light near the band edge) and especially in tandem sub cells.

Turning to FREAs, Huang et al. modified Y6 as an ETL, achieving over 25% PCE in fullerene-free perovskite solar cells, as shown in Figure 4A,B. This work offers the highest PCE for fullerene-free devices to date, bringing hope that the efficiency gap with fullerene devices will soon be closed. It should be noted, however, that a small molecule (bathocuproine, BCP)—previously shown to exhibit a low *G*_c_ comparable to fullerenes—was employed in combination with Y6. Although the *G*_c_ of Y6 has not been measured, it is also a small molecule, and there are therefore two potential sources of fragility in this device stack. The work could be further improved by benchmarking the stability against devices with C_60_. Despite the commendable efficiency, this approach moves backward on the cost challenge, since Y6 is roughly 25 times more expensive than fullerenes at lab scale.^86^ In one report, Y6 production requires 15 synthetic steps while another report found a yield of just 12%.^87–89^ In addition, Y6 and other FREA have the end group acceptor connect to the core through a double bond that is highly polarized and susceptible to nucleophilic attack. The long-term stability of such molecules that could come into contact with halide anions is a factor that needs to be explored carefully before such materials could be considered for commercial applications.

**Figure 4:**
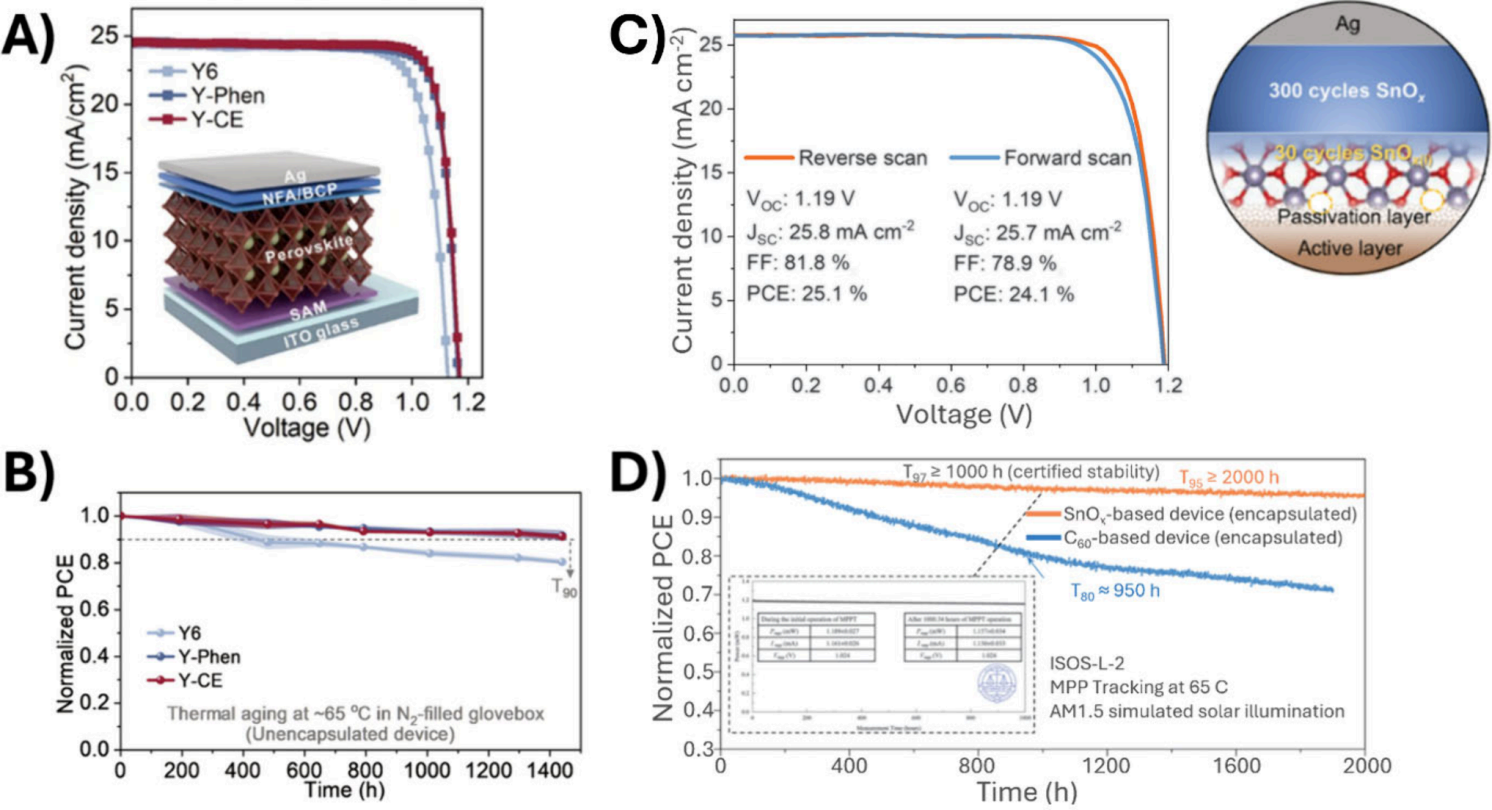
Recent innovations in fullerene-free PSC. (A, B) Efficiency and stability of perovskite cell with a Y6-derived small molecule ETL. Reproduced with permission from ref 26. Copyright 2024 Science. (C, D) Efficiency and stability of perovskite cell with hybrid ETL (PDINN-ALD SnO_*x*_) showing substantial improvement when benchmarked against C_60_ control device. Adapted with permission from ref 51. Copyright 2024 Nature.

In contrast to Y6, an NDI polymer used in a cell with efficiency near 20% has a reported yield of 70% with only 3 synthetic steps.^18^ We believe NDIs may be one of the most promising candidates for incorporation into commercial scale, durable perovskite PV for several reasons. First, NDIs offer simple synthesis and purification, with costs often below $10 g^–1^. Mobilities of solution-processed NDI films reach 10^–4^ cm^2^/(V s), comparable to thermally evaporated C_60_ films, and the thermal decomposition of NDI’s is well above the operating temperature for PV. In general, simple NDI materials are both thermally and photochemically stable, and chemically resistant in undoped forms.^90^ With organic materials, it is relatively easy to tune the LUMO energy over a broad range. (Figure 3B), offering potentially higher efficiencies for tandem perovskites. NDIs, unlike PDIs and Y6 derivatives, can also exhibit minimal absorption across the visible spectrum, reducing parasitic absorption.^70^ Finally, NDI polymers, such as that in Figure 3B, also provide unparalleled *G*_c_ among ETLs, a critical step toward multidecadal PV lifetimes. A potential limitation of NDI polymers and small molecules is that, while it is easy to lower the LUMO by substitution of the NDI with acceptors, substation of the NDIs with donors leads to only modest changes of the LUMO. However, substitution with donors significantly increases the energy of the HOMO (i.e., they lower the ionization energy) and thus decreases the optical gap, introducing parasitic absorption in visible. In the most successful demonstration of a rylene alternative to fullerenes, Gao et al. replaced C_60_ with ∼2 nm thick PDINN, a perylene diimide (PDI) derived molecule, demonstrating successful ALD SnO_*x*_ in PSC without a fullerene interlayer for the first time, as shown in Figure 4C,D. The 25.1% efficient solar cells offer greatly improved ambient stability relative to devices with C_60_, which is attributed to the permeability of C_60_, both to atmospheric O_2_, H_2_O, and egressing halide species. Despite its success, there are two challenges to this approach.

First, the spin-coated PDINN layer thickness of ∼2 nm is difficult to scale to large areas using solution processing techniques since float glass tolerances (±0.2–0.5 mm) are on the order of slot dye and blade coater gap heights.^91^ Thermal evaporation of the PDINN or other buffering interlayers for ALD SnO_*x*_ could accelerate adoption. There are few reports of vapor deposited NDI’s and further investigation needs to determine whether a PV relevant PDI or NDI can be vapor deposited without decomposition.^92–94^ Charge transport through NDI films is also anisotropic, in contrast to isotropic transport through symmetric C_60_ molecules, making the device performance more sensitive to the deposition conditions. The deposition solvent plays a crucial role in molecular reorientation; in one study, aggregation-prone solvents for a polymer NDI produced films that had 2 orders of magnitude higher mobility than in solvents that prevented aggregation.^95^ Potentially, a vapor deposited NDI, as recently demonstrated in n-i-p devices,^96^ could be combined with subsequent solvent-vapor annealing to enable uniform coverage and improved transport. NDI’s with a vinyl group addition have also been vapor deposited with an electron beam, polymerizing during deposition.^97^

In conclusion, as PSCs move closer to commercialization, finding alternatives to fullerenes becomes ever more important to ensure the long-term mechanical integrity of perovskite modules, and to improve their efficiency and reproducibility. We emphasize that the evaluated nonfullerene ETLs and their corresponding stacks—including polymer-based NDI, hybrid, and inorganic architectures employing ALD with either nanoparticles or a PDINN layer—exhibited fracture energies exceeding 1 J·m^–2^, a threshold indicative of mechanical robustness for PSCs. We can hasten the widespread adoption of fullerene-free cells by certifying PCEs (or providing reverse and forward scans and SPO), benchmarking stability against fullerene devices using International Summit on Organic Photovoltaic Stability (ISOS) protocols, and by reproducing architectures across multiple laboratories. While OPV materials with known performance are tempting to adopt into perovskite cells, we believe there will be long-term benefits for designing ETLs for perovskite cells, avoiding the unneeded synthetic complexity and costs associated with OPV materials, as well as significant absorption in the visible-NIR and potential chemical instabilities.

Despite their flaws, fullerenes remain an entrenched feature of perovskite solar cells because they provide reproducible, efficient cells with reasonable lifetimes under stress tests that avoid thermal cycling. Recent reports of fullerene-free cells with efficiency above 25% have given a glimpse into a fullerene-free future for perovskite cells, but these architectures have yet to be reproduced after their publication, and the field is far from making fullerene-free architectures the de facto standard. OPV’s reliance on fullerenes for two decades after their shortcomings were widely known stands as a cautionary tale against one-off demonstrations. It is incumbent on the perovskite community to overcome the challenges of fullerene-free devices.

## Supplementary Material



## Notes

Methods and expanded figures of merit from literature (PDF)
