# Supplementary material for: Toward Fullerene-Free PIN Perovskite Solar Cells
Source: ACS Energy Lett. 2025 Nov 18;10(12):6417–27. doi: 10.1021/acsenergylett.5c02987 (PMC12706837; doi:10.1021/acsenergylett.5c02987)
Supplement: Supplementary file 1 [file nz5c02987_si_001.pdf]

Supplemental Information

Towards Fullerene-free PIN Perovskite Solar Cells

Kelly Schutt,[1,+] Melissa Davis,[1,+] Muzhi Li,[2,+] Samuel A. Johnson,[2] Daniel Martinez[4], Jochen Titus[4], Tomas Leijtens[4], Blake Martin[5], Michael D. McGehee[3], Seth R. Marder[3], Nicholas Rolston[2,], Joseph M. Luther[*1,3]*

[1] National Renewable Energy Laboratory, Golden, CO 80401, United States
[2]Materials Science and Engineering, Fulton Schools of Engineering, Arizona State University, Tempe, AZ 85287, United States
[3] Renewable and Sustainable Energy Institute, University of Colorado, Boulder, CO 80309, United States
[4] Swift Solar, 981 Bing Street, San Carlos CA 94070, United States
[5] Sofab Inks, 11351 Decimal Drive, Louisville, KY 40299, United States
[+] Indicates equal contribution
* Corresponding author's email: nicholas.rolston@asu.edu and joey.luther@nrel.gov

Methods

Substrates were cleaned by scrubbing with a detergent solution and then ultrasonication in DI water, acetone, and IPA for 2 minutes in each solvent. After a 15-minute ultraviolet ozone treatment on the bare substrates, the substrates were coated in sputtered NiO_x and then annealed in air for 10 minutes at 300 °C. The substrates were then transferred into a nitrogen glovebox where the hole transport material and active layer were deposited. The active layer with composition $Cs_{0.05}MA_{0.16}FA_{0.79}Pb(I_{0.84}Br_{0.16})_3$ was then deposited by distributing 30 uL of precursor ink onto the ITO substrate and spreading to cover the entire

surface and spin coating. The precursor was nitrogen quenched during spin coating using a 3/8" inner diameter stainless steel tube positioned vertically 5 mm over the center of the substrate. Nitrogen pressure was approximately 100 psig with a flow of 3.2 SCFM. PCBM was spin coated at 20 mg/mL at 2k rpm for 30 s and then annealed for 5 min at 100 °C. C_{60} was thermally evaporated at 0.5-2.0 Å/s at a pressure of ~10^{-6} torr to reach a thickness of 25 nm. BCP was evaporated at 0.1 Å/s at a pressure of ~10^{-6} torr to reach a thickness of 6 nm. NDI's were dissolved in chlorobenzene at a concentration of 5 mg/mL and spin coated at 2k rpm for 30 s, then annealed for 5 min at 100 °C. 4F-PEAI was prepared at 2 mg/mL in 200:1 IPA:DMF and dynamically spin coated at 2k rpm for 30 s, then annealed 5 min at 100 °C. PDINN was prepared at 2 mg/mL in trifluoroethanol, dynamically spin coated at 5k rpm for 30 s, then annealed at 100 °C for 10 min. ALD SnO_x was grown in a Beneq TFS-200 ALD system by reacting de-ionized water and tetrakis(dimethylamino) tin(IV) at a reactor temperature of 90 °C, as described in previous work.[1]

Double cantilever beam (DCB) samples adopted a structure of glass/ITO/HTL/perovskite/ETL/Ag/epoxy/glass. In the cases where a half stack was tested and no ETL was used (perovskite or 4F-PEAI on perovskite), a PMMA layer (~800 nm) was spin-coated onto the perovskite for protection against the Ag, using a 0.1 g/mL PMMA solution in chlorobenzene at 3000 rpm for 30 s. The epoxy used was Loctite epoxy instant mix 5min, which was applied to a bare glass superstrate and bonded to the glass substrate deposited with perovskite stacks to form DCB samples. Specific details of the testing method can be found

elsewhere.[2] Briefly, a pre-crack was introduced into the DCB sample along the orientation of the crack propagation by inserting the tip of a razor blade into the DCB sample. The resulting pre-crack can protect the DCB sample from excessive tensile loads and initiates crack propagation. By using the delaminator system (DTS, USA), the pre-cracked DCB sample was mounted and loaded in tension at a displacement rate of 1 μm/s. When a clear drop occurred in the load-displacement curve, the DCB sample was partially unloaded to calculate the compliance and then loaded again to gradually propagate the crack until a complete separation was achieved for the two bonded glass substrates, suggesting the completion of the measurement. In the measurement, the load (P) – displacement (Δ) curves were continuously recorded and used to extract the fracture energy (G_c), which can be calculated from Eq. (1):

$$G_C = \frac{12P_c^2 a^2}{B^2 E' h^3}\left(1 + 0.64\frac{h}{a}\right)^2 \tag{1}$$

where P_c is the critical load that deviates from the linearity in the P-Δ curve during the loading cycle; a is the crack length; B and h are the width and half height of the sample, respectively; and E' is the plane-strain elastic modulus of the substrate. The crack length was estimated using the compliance method in Eq. (2):

$$a = \left(\frac{d\Delta}{dP} * \frac{BE' h^3}{8}\right)^{\frac{1}{3}} - 0.64 * h \tag{2}$$

All G_c testing was carried out in laboratory air environment at ~20 °C and ~20% R.H.

Material/Class	*Best Perovskite Cell Efficiency (%)*	*Permeability/WVTR (g/m²-day)*	*Mobility (cm²/Vs)*	*Transmittance (%T)*	*Cost (g/$)*	*Reproducibility (# publications)*
Fullerenes	26.7 [3]	5.0×10^{-2} (100 nm C_{60}) [4]	6.5×10^{-4} (C_{60}) [5]<br>2.5×10^{-4} (PCBM) [6]	~88% at 500 nm (25 nm C_{60})[7]	5.7×10^{-3} (Sigma Aldrich, C_{60}) [8]	9,830 results
Small Molecules	25.59 [9]	6.9 (unencapsulated organic solar cell) [10]	2.5×10^{-4} (PDINN)[11]	~98% 400-800 nm (PDINN ETL)[12]	3.9×10^{-4} (Y6, Ossila) [13]<br>0.1 (n-type NDI) [14]<br>0.02 (NDI ETL) [15]	6<br>(Fig 1C / Table S3)
Polymers	26.0 [16]	~1.3 (75 µm PET) [17]<br>>10 (polyamide, polylactic acid) [18]	4.8×10^{-4}, 0.46, 4.87 (NDI polymers) [6]	>90% 400-800 nm (NDI polymer) [19]	0.015 (NDI polymer, computed from SI synthesis materials) [19]	4<br>(Fig 1C / Table S3)

Nanoparticles	20.4 [20]	9.5×10^{-3} (sol-gel AlO_x on PEN) [21]<br>3.9×10^{-2} (TiO_x on PEN) [22]	4.2×10^{2} (SnO_2 ETL) [23]	~95% 400-800 nm (SnO_2 ETL) [24]	0.079 (Sigma Aldrich, SnO_2 nanopowder) [25]	4<br>(Fig 1C / Table S3)
ALD/Vapor	13.3% (CVD TiO_x) [26]<br>23.0% (np + ALD) [27]	10^{-5} (25 nm ALD Al_2O_3 on PEN) [1]	11-36 (ALD SnO_x) [28,29]	~90% 400-800 nm (40 nm ALD SnO_2)[30]	0.2<br>(TDMASn) [31]	3<br>(Fig 1C / Table S3)
Hybrid	25.1 [32]	10^{-5} (25 nm ALD Al_2O_3 on PEN) [1]	2.5×10^{-4} (PDINN)[11]	~98% 400-800 nm (PDINN ETL) [12]<br>~90% 400-800 nm (40 nm ALD SnO_2) [30]	9.5×10^{-4} (PDINN, Ossila) [33]<br>0.2<br>(TDMASn) [31]	2<br>(Fig 1C / Table S3)

Table S1: Multi-property comparison of ETL materials. Data collected for ETLs in p-i-n perovskite solar cells when available. The number of publications for fullerenes is reported from Google Scholar results for the query “perovskite solar AND (pcbm OR c60) AND p-i-n)”.

Year	PCE	Reference
2016	18.72	[34]
2017	20.59	[35]
2020	23.0	[36]
2021	23.6	[37]
2022	24.3	[38]
2022	25.3	[39]
2023	26.4	[40]
2025	26.7	[3]

Table S2: Best efficiencies for published p-i-n, fullerene containing perovskite solar cells

Year	PCE	ETL	Reference
2016	11.06	F16CuPc	[41]
2016	16.7	NDI derivative polymer	[42]
2017	19.6	NDI small molecule	[43]
2018	15.28	PDIN small molecule	[44]
2018	20.2	NDI small molecule	[45]
2019	20.5	NDI small molecule	[46]
2020	13.3	TiOx CVD	[26]
2020	19.2	Perylene derivative	[47]
2020	20.8	NDI, PDI derivatives polymer	[48]
2020	19.9	NDI small molecule	[49]
2024	18.9	SnO2	[50]
2024	20.4	SnO2	[20]
2024	25.1	PDINN-SnO2	[32]
2024	23.0	SnO2 (nanoparticle + ALD)	[27]
2025	25.59	Y6 derivative	[9]
2026	26.0	Polymer	[16]

Table S3: Efficiencies for published p-i-n, fullerene-free perovskite solar cells

Material	CB/LUMO	VB / HOMO	Reference
1.2 eV perovskite	-4.0	-5.27	[51,52]
1.8 eV perovskite	-3.8	-5.6	[51,53]
1.5 eV perovskite	-4	-5.4	[54]
C60	-4.5	-6.2	[55]
PCBM	-4.2	-6	[56]
SnOx	-4.46 to -4.05	-7.8	[57]

Table S4: Energy levels of perovskites and ETL's

Material and Interface	***Fracture Energy ($J \cdot m^{-2}$)***
Perovskite	1.72-2.58
4F-PEAI+Perovskite	3.89-4.71
$C_{60/BCP}$	0.17-0.29
PCBM	0.27-0.38
BCP	0.43-0.51
NDI	0.24-0.33
NDI-n	4.35-5.04
Nano SnO_x	0.10-0.17
ALD SnO_x	0.47-0.63
Nano + ALD	1.00-1.69
Sputter ITO	0.98-1.37
PDINN + ALD SnO_x	1.29-1.70

Table S5: Fracture energy (G_c) ranges of the measured materials in Fig. 3B

REFERENCES

(1) Johnson, S. A.; White, K. P.; Tong, J.; You, S.; Magomedov, A.; Larson, B. W.; Morales, D.; Bramante, R.; Dunphy, E.; Tirawat, R.; Perkins, C. L.; Werner, J.; Lahti, G.; Velez, C.; Toney, M. F.; Zhu, K.; McGehee, M. D.; Berry, J. J.; Palmstrom, A. F. Improving the Barrier Properties of Tin Oxide in Metal Halide Perovskite Solar Cells Using Ozone to Enhance Nucleation. *Joule* **2023**, *7* (12), 2873–2893. https://doi.org/10.1016/j.joule.2023.10.009.

(2) Bhosale, S. V.; Kobaisi, M. A.; Jadhav, R. W.; Morajkar, P. P.; Jones, L. A.; George, S. Naphthalene Diimides: Perspectives and Promise. *Chem. Soc. Rev.* **2021**, *50* (17), 9845–9998. https://doi.org/10.1039/D0CS00239A.

(3) Gao, D.; Li, B.; Sun, X.; Liu, Q.; Zhang, C.; Qian, L.; Yu, Z.; Li, X.; Wu, X.; Liu, B.; Wang, N.; Vanin, F.; Xia, X.; Gong, J.; Li, N.; Zeng, X. C.; Li, Z.; Zhu, Z. High-Efficiency Perovskite Solar Cells Enabled by Suppressing Intermolecular Aggregation in Hole-Selective Contacts. *Nat. Photon.* **2025**, 1–8. https://doi.org/10.1038/s41566-025-01725-x.

(4) *Device-Like Electrical Calcium Corrosion Test for WVTR Measurements of Ultra-Barriers - The Society of Vacuum Coaters*. https://www.svc.org/digital-library/web-tech-roll-to-roll-coatings-for-high-end-applications/device-like-electrical-calcium-corrosion-test-for-wvtr-measurements-of-ultra-barriers/ (accessed 2025-09-15).

(5) Xing, Z.; Ma, S.; Chen, B.-W.; An, M.; Fan, A.; Hu, X.; Wang, Y.; Deng, L.-L.; Huang, Q.; Kanda, H.; Al-Amri, F. G.; Pozzi, G.; Zhang, Y.; Xia, J.; Wu, J.; Guo, X.; Nazeeruddin, M. K. Solubilizing and Stabilizing C60 with N-Type Polymer Enables Efficient Inverted Perovskite Solar Cells. *Joule* **2025**, *9* (4), 101817. https://doi.org/10.1016/j.joule.2024.101817.

(6) Jameel, M. A.; Yang, T. C.-J.; Wilson, G. J.; Evans, R. A.; Gupta, A.; Langford, S. J. Naphthalene Diimide-Based Electron Transport Materials for Perovskite Solar Cells. *J. Mater. Chem. A* **2021**, *9* (48), 27170–27192. https://doi.org/10.1039/D1TA08424K.

(7) Faiman, D.; Goren, S.; Katz, E. A.; Koltun, M.; Melnik, N.; Shames, A.; Shtutina, S. Structure and Optical Properties of C60 Thin Films. *Thin Solid Films* **1997**, *295* (1), 283–286. https://doi.org/10.1016/S0040-6090(96)09043-8.

(8) *Fullerene-C60 99.5 99685-96-8*. https://www.sigmaaldrich.com/US/en/product/aldrich/379646 (accessed 2025-09-15).

(9) Huang, X.; Xia, D.; Xie, Q.; Wang, D.; Li, Q.; Zhao, C.; Yin, J.; Cao, F.; Su, Z.; Zeng, Z.; Jiang, W.; Kaminsky, W.; Liu, K.; Lin, F. R.; Feng, Q.; Wu, B.; Tsang, S.-W.; Lei, D.; Li, W.; Jen, A. K.-Y. Supramolecular Force-Driven Non-Fullerene Acceptors as an Electron-Transporting Layer for Efficient Inverted Perovskite Solar Cells. *Nat Commun* **2025**, *16* (1), 1626. https://doi.org/10.1038/s41467-025-56060-0.

(10) Hermenau, M.; Schubert, S.; Klumbies, H.; Fahlteich, J.; Müller-Meskamp, L.; Leo, K.; Riede, M. The Effect of Barrier Performance on the Lifetime of Small-Molecule Organic

Solar Cells. *Solar Energy Materials and Solar Cells* **2012**, *97*, 102–108. https://doi.org/10.1016/j.solmat.2011.09.026.

(11) Zhou, D.; Han, L.; Hu, L.; Yang, S.; Shen, X.; Li, Y.; Tong, Y.; Wang, F.; Li, Z.; Chen, L. Bay-Functionalized Perylene Diimide Derivative Cathode Interfacial Layer for High-Performance Organic Solar Cells. *ACS Appl. Mater. Interfaces* **2023**, *15* (6), 8367–8376. https://doi.org/10.1021/acsami.2c22069.

(12) Zhang, G.; Wang, L.; Zhao, C.; Wang, Y.; Hu, R.; Che, J.; He, S.; Chen, W.; Cao, L.; Luo, Z.; Qiu, M.; Li, S.; Zhang, G. Efficient All-Polymer Solar Cells Enabled by Interface Engineering. *Polymers* **2022**, *14* (18), 3835. https://doi.org/10.3390/polym14183835.

(13) *Y6, BTP-4F*. Ossila. https://www.ossila.com/products/y6 (accessed 2025-09-15).

(14) Giri, I.; Chhetri, S.; P, J. J.; Mondal, M.; Bikash Dey, A.; K. Vijayaraghavan, R. Engineered Solid-State Aggregates in Brickwork Stacks of n-Type Organic Semiconductors: A Way to Achieve High Electron Mobility. *Chemical Science* **2024**, *15* (25), 9630–9640. https://doi.org/10.1039/D4SC02339K.

(15) Li, X.; Wang, W.; Huang, P.; Yang, L.; Hu, J.; Wei, K.; Gao, L.; Jiang, Y.; Sun, K.; Du, G.; Cai, X.; Liu, C.; Tang, W.; Zhang, J. Fluorinated Naphthalene Diimides as Buried Electron Transport Materials Achieve Over 23% Efficient Perovskite Solar Cells. *Advanced Science* **2024**, *11* (36), 2403735. https://doi.org/10.1002/advs.202403735.

(16) Feng, K.; Wang, G.; Lian, Q.; Gámez-Valenzuela, S.; Li, B.; Ding, R.; Yang, W.; Wang, K.; Zeng, J.; Zhang, Y.; Jeong, S. Y.; Xu, B.; Ho-Baillie, A.; Woo, H. Y.; Facchetti, A.; Guo, X. Non-Fullerene Electron-Transporting Materials for High-Performance and Stable Perovskite Solar Cells. *Nat. Mater.* **2025**, *24* (5), 770–777. https://doi.org/10.1038/s41563-025-02163-4.

(17) Jarvis, K. L.; Evans, P. J.; Cooling, N. A.; Vaughan, B.; Habsuda, J.; Belcher, W. J.; Bilen, C.; Griffiths, G.; Dastoor, P. C.; Triani, G. Comparing Three Techniques to Determine the Water Vapour Transmission Rates of Polymers and Barrier Films. *Surfaces and Interfaces* **2017**, *9*, 182–188. https://doi.org/10.1016/j.surfin.2017.09.009.

(18) Sängerlaub, S.; Schmid, M.; Müller, K. Comparison of Water Vapour Transmission Rates of Monolayer Films Determined by Water Vapour Sorption and Permeation Experiments. *Food Packaging and Shelf Life* **2018**, *17*, 80–84. https://doi.org/10.1016/j.fpsl.2018.06.004.

(19) Shi, Y.; McCarthy, D. P.; Lungwitz, D.; Jiang, F.; Taddei, M.; Contreras, H.; Lin, Y.; Mohapatra, A. A.; Tang, K.; Zhang, Y.; Barlow, S.; Kahn, A.; Marder, S. R.; Ginger, D. S. Photo-Crosslinkable Naphthalene Diimide Polymer for Solution-Processed n–i–p Perovskite Solar Cells. *Chem. Mater.* **2024**, *36* (2), 795–802. https://doi.org/10.1021/acs.chemmater.3c02295.

(20) Chapagain, S.; Armstrong, P. J.; Panta, R.; Acharya, N.; Druffel, T.; Grapperhaus, C. A. Expanding the Solvent Diversity and Perovskite Compatibility of SnO2 Inks That Are Directly Deposited on Perovskite Layers. *iScience* **2024**, *27* (10), 110964. https://doi.org/10.1016/j.isci.2024.110964.

(21) Park, S.; Jeong, Y. J.; Baek, Y.; Kim, L. H.; Jang, J. H.; Kim, Y.; An, T. K.; Nam, S.; Kim, S. H.; Jang, J.; Park, C. E. Reduced Water Vapor Transmission Rates of Low-Temperature

Solution-Processed Metal Oxide Barrier Films *via* Ultraviolet Annealing. *Applied Surface Science* **2017**, *414*, 262–269. https://doi.org/10.1016/j.apsusc.2017.04.100.

(22) Park, S.; Kim, L. H.; Jeong, Y. J.; Kim, K.; Park, M.; Baek, Y.; An, T. K.; Nam, S.; Jang, J.; Park, C. E. Reduced Water Vapor Transmission Rates of Low-Temperature-Processed and Sol-Gel-Derived Titanium Oxide Thin Films on Flexible Substrates. *Organic Electronics* **2016**, *36*, 133–139. https://doi.org/10.1016/j.orgel.2016.05.042.

(23) Ke, W.; Zhao, D.; Cimaroli, A. J.; Grice, C. R.; Qin, P.; Liu, Q.; Xiong, L.; Yan, Y.; Fang, G. Effects of Annealing Temperature of Tin Oxide Electron Selective Layers on the Performance of Perovskite Solar Cells. *J. Mater. Chem. A* **2015**, *3* (47), 24163–24168. https://doi.org/10.1039/C5TA06574G.

(24) Schutt, K.; Nayak, P. K.; Ramadan, A. J.; Wenger, B.; Lin, Y.-H.; Snaith, H. J. Overcoming Zinc Oxide Interface Instability with a Methylammonium-Free Perovskite for High-Performance Solar Cells. *Advanced Functional Materials* **2019**, *29* (47), 1900466. https://doi.org/10.1002/adfm.201900466.

(25) *Tin(IV) oxide nanopowder, avg. part. size = 100nm 18282-10-5*. https://www.sigmaaldrich.com/US/en/product/aldrich/549657 (accessed 2025-09-16).

(26) Chen, J. P.; Hilt, F.; Rolston, N.; Dauskardt, R. H. Scalable Open-Air Deposition of Compact ETL TiOx on Perovskite for Fullerene-Free Solar Cells. *J. Mater. Chem. A* **2020**, *8* (43), 22858–22866. https://doi.org/10.1039/D0TA08554E.

(27) Fei, F.; Liao, Y.; Xu, Y.; Wang, S.; Li, L.; Dong, X.; Zhou, X.; Gao, J.; Wang, K.; Yuan, N.; Ding, J. Stable Inverted Perovskite Solar Cells with Efficiency over 23.0% via Dual-Layer SnO2 on Perovskite. *ACS Appl. Mater. Interfaces* **2024**, *16* (19), 24760–24770. https://doi.org/10.1021/acsami.4c02559.

(28) Kuang, Y.; Zardetto, V.; van Gils, R.; Karwal, S.; Koushik, D.; Verheijen, M. A.; Black, L. E.; Weijtens, C.; Veenstra, S.; Andriessen, R.; Kessels, W. M. M.; Creatore, M. Low-Temperature Plasma-Assisted Atomic-Layer-Deposited SnO2 as an Electron Transport Layer in Planar Perovskite Solar Cells. *ACS Appl Mater Interfaces* **2018**, *10* (36), 30367–30378. https://doi.org/10.1021/acsami.8b09515.

(29) Nguyen, V. H.; Akbari, M.; Sekkat, A.; Ta, H. T. T.; Resende, J.; Jiménez, C.; Musselman, K. P.; Muñoz-Rojas, D. Atmospheric Atomic Layer Deposition of SnO2 Thin Films with Tin(II) Acetylacetonate and Water. *Dalton Trans.* **2022**, *51* (24), 9278–9290. https://doi.org/10.1039/D2DT01427K.

(30) Choi, D.; Park, J.-S. Highly Conductive SnO2 Thin Films Deposited by Atomic Layer Deposition Using Tetrakis-Dimethyl-Amine-Tin Precursor and Ozone Reactant. *Surface and Coatings Technology* **2014**, *259*, 238–243. https://doi.org/10.1016/j.surfcoat.2014.02.012.

(31) Cordell, J. J.; Woodhouse, M.; Warren, E. L. Technoeconomic Analysis of Perovskite/Silicon Tandem Solar Modules. *Joule* **2025**, *9* (2), 101781. https://doi.org/10.1016/j.joule.2024.10.013.

(32) Gao, D.; Li, B.; Liu, Q.; Zhang, C.; Yu, Z.; Li, S.; Gong, J.; Qian, L.; Vanin, F.; Schutt, K.; Davis, M. A.; Palmstrom, A. F.; Harvey, S. P.; Long, N. J.; Luther, J. M.; Zeng, X. C.; Zhu,

Z. Long-Term Stability in Perovskite Solar Cells through Atomic Layer Deposition of Tin Oxide. *Science* **2024**, *386* (6718), 187–192. https://doi.org/10.1126/science.adq8385.
(33) *PDINN*. Ossila. https://www.ossila.com/products/pdinn (accessed 2025-09-16).
(34) Chen, K.; Hu, Q.; Liu, T.; Zhao, L.; Luo, D.; Wu, J.; Zhang, Y.; Zhang, W.; Liu, F.; Russell, T. P.; Zhu, R.; Gong, Q. Charge-Carrier Balance for Highly Efficient Inverted Planar Heterojunction Perovskite Solar Cells. *Advanced Materials* **2016**, *28* (48), 10718–10724. https://doi.org/10.1002/adma.201604048.
(35) Zheng, X.; Chen, B.; Dai, J.; Fang, Y.; Bai, Y.; Lin, Y.; Wei, H.; Zeng, X. C.; Huang, J. Defect Passivation in Hybrid Perovskite Solar Cells Using Quaternary Ammonium Halide Anions and Cations. *Nat Energy* **2017**, *2* (7), 17102. https://doi.org/10.1038/nenergy.2017.102.
(36) Zheng, X.; Hou, Y.; Bao, C.; Yin, J.; Yuan, F.; Huang, Z.; Song, K.; Liu, J.; Troughton, J.; Gasparini, N.; Zhou, C.; Lin, Y.; Xue, D.-J.; Chen, B.; Johnston, A. K.; Wei, N.; Hedhili, M. N.; Wei, M.; Alsalloum, A. Y.; Maity, P.; Turedi, B.; Yang, C.; Baran, D.; Anthopoulos, T. D.; Han, Y.; Lu, Z.-H.; Mohammed, O. F.; Gao, F.; Sargent, E. H.; Bakr, O. M. Managing Grains and Interfaces via Ligand Anchoring Enables 22.3%-Efficiency Inverted Perovskite Solar Cells. *Nat Energy* **2020**, *5* (2), 131–140. https://doi.org/10.1038/s41560-019-0538-4.
(37) Chen, S.; Dai, X.; Xu, S.; Jiao, H.; Zhao, L.; Huang, J. Stabilizing Perovskite-Substrate Interfaces for High-Performance Perovskite Modules. *Science* **2021**, *373* (6557), 902–907. https://doi.org/10.1126/science.abi6323.
(38) *Damp heat–stable perovskite solar cells with tailored-dimensionality 2D/3D heterojunctions | Science*. https://www.science.org/doi/10.1126/science.abm5784 (accessed 2025-09-04).
(39) Jiang, Q.; Tong, J.; Xian, Y.; Kerner, R. A.; Dunfield, S. P.; Xiao, C.; Scheidt, R. A.; Kuciauskas, D.; Wang, X.; Hautzinger, M. P.; Tirawat, R.; Beard, M. C.; Fenning, D. P.; Berry, J. J.; Larson, B. W.; Yan, Y.; Zhu, K. Surface Reaction for Efficient and Stable Inverted Perovskite Solar Cells. *Nature* **2022**, *611* (7935), 278–283. https://doi.org/10.1038/s41586-022-05268-x.
(40) Liu, C.; Yang, Y.; Chen, H.; Xu, J.; Liu, A.; Bati, A. S. R.; Zhu, H.; Grater, L.; Hadke, S. S.; Huang, C.; Sangwan, V. K.; Cai, T.; Shin, D.; Chen, L. X.; Hersam, M. C.; Mirkin, C. A.; Chen, B.; Kanatzidis, M. G.; Sargent, E. H. Bimolecularly Passivated Interface Enables Efficient and Stable Inverted Perovskite Solar Cells. *Science* **2023**, *382* (6672), 810–815. https://doi.org/10.1126/science.adk1633.
(41) Jin, F.; Liu, C.; Hou, F.; Song, Q.; Su, Z.; Chu, B.; Cheng, P.; Zhao, H.; Li, W. Hexadecafluorophthalocyaninatocopper as an Electron Conductor for High-Efficiency Fullerene-Free Planar Perovskite Solar Cells. *Solar Energy Materials and Solar Cells* **2016**, *157*, 510–516. https://doi.org/10.1016/j.solmat.2016.07.037.
(42) Sun, C.; Wu, Z.; Yip, H.-L.; Zhang, H.; Jiang, X.-F.; Xue, Q.; Hu, Z.; Hu, Z.; Shen, Y.; Wang, M.; Huang, F.; Cao, Y. Amino-Functionalized Conjugated Polymer as an Efficient Electron Transport Layer for High-Performance Planar-Heterojunction Perovskite Solar

Cells. *Advanced Energy Materials* **2016**, *6* (5), 1501534. https://doi.org/10.1002/aenm.201501534.

(43) Heo, J. H.; Lee, S.-C.; Jung, S.-K.; Kwon, O.-P.; Im, S. H. Efficient and Thermally Stable Inverted Perovskite Solar Cells by Introduction of Non-Fullerene Electron Transporting Materials. *J. Mater. Chem. A* **2017**, *5* (39), 20615–20622. https://doi.org/10.1039/C7TA06900F.

(44) Miao, J.; Hu, Z.; Liu, M.; Umair Ali, M.; Goto, O.; Lu, W.; Yang, T.; Liang, Y.; Meng, H. A Non-Fullerene Small Molecule Processed with Green Solvent as an Electron Transporting Material for High Efficiency p-i-n Perovskite Solar Cells. *Organic Electronics* **2018**, *52*, 200–205. https://doi.org/10.1016/j.orgel.2017.10.028.

(45) Jung, S.-K.; Heo, J. H.; Lee, D. W.; Lee, S.-C.; Lee, S.-H.; Yoon, W.; Yun, H.; Im, S. H.; Kim, J. H.; Kwon, O.-P. Nonfullerene Electron Transporting Material Based on Naphthalene Diimide Small Molecule for Highly Stable Perovskite Solar Cells with Efficiency Exceeding 20%. *Advanced Functional Materials* **2018**, *28* (20), 1800346. https://doi.org/10.1002/adfm.201800346.

(46) Jung, S.-K.; Heo, J. H.; Lee, D. W.; Lee, S.-H.; Lee, S.-C.; Yoon, W.; Yun, H.; Kim, D.; Kim, J. H.; Im, S. H.; Kwon, O.-P. Homochiral Asymmetric-Shaped Electron-Transporting Materials for Efficient Non-Fullerene Perovskite Solar Cells. *ChemSusChem* **2019**, *12* (1), 224–230. https://doi.org/10.1002/cssc.201802234.

(47) Xie, C.; Zhou, C.; Yang, B.; Shen, L.; Ke, L.; Ding, L.; Yuan, Y. Silicon Phthalocyanine Passivation for Fullerene-Free Perovskite Solar Cells with Efficient Electron Extraction. *Appl. Phys. Express* **2019**, *12* (6), 064006. https://doi.org/10.7567/1882-0786/ab1fb6.

(48) Shi, Y.; Chen, W.; Wu, Z.; Wang, Y.; Sun, W.; Yang, K.; Tang, Y.; Woo, H. Y.; Zhou, M.; Djurišić, A. B.; He, Z.; Guo, X. Imide-Functionalized Acceptor–Acceptor Copolymers as Efficient Electron Transport Layers for High-Performance Perovskite Solar Cells. *J. Mater. Chem. A* **2020**, *8* (27), 13754–13762. https://doi.org/10.1039/D0TA03548C.

(49) Jung, S.-K.; Heo, J. H.; Oh, B. M.; Lee, J. B.; Park, S.-H.; Yoon, W.; Song, Y.; Yun, H.; Kim, J. H.; Im, S. H.; Kwon, O.-P. Chiral Stereoisomer Engineering of Electron Transporting Materials for Efficient and Stable Perovskite Solar Cells. *Advanced Functional Materials* **2020**, *30* (13), 1905951. https://doi.org/10.1002/adfm.201905951.

(50) Kim, S. Y.; Woo, M. Y.; Jeong, M. J.; Jeon, S. W.; Ahn, J. W.; Park, J. H.; Kim, C. Y.; Kim, D. H.; Oh, O. J.; Yu, G.; Lee, S.; Kim, C.; Kim, D. H.; Noh, J. H. Fullerene-Free p–i–n Perovskite Solar Cells: Direct Deposition of Tin Oxide on Perovskite Layer Using Ligand Bridges. *Advanced Energy Materials* **2024**, *14* (48), 2402433. https://doi.org/10.1002/aenm.202402433.

(51) Lin, R.; Wang, Y.; Lu, Q.; Tang, B.; Li, J.; Gao, H.; Gao, Y.; Li, H.; Ding, C.; Wen, J.; Wu, P.; Liu, C.; Zhao, S.; Xiao, K.; Liu, Z.; Ma, C.; Deng, Y.; Li, L.; Fan, F.; Tan, H. All-Perovskite Tandem Solar Cells with 3D/3D Bilayer Perovskite Heterojunction. *Nature* **2023**, *620* (7976), 994–1000. https://doi.org/10.1038/s41586-023-06278-z.

(52) Yang, X.; Zhou, W.; He, Y.; Sun, Z.; Zeng, Q.; Yan, H.; Zheng, Z.; Chen, X.; Tang, Z.; Zhang, J. Design of Efficient Inverted NiOX-Based Three-Terminal Back-Contact All

Perovskite Tandem Solar Cells. *Advanced Functional Materials* **2024**, *34* (29), 2401508. https://doi.org/10.1002/adfm.202401508.
(53) Yang, X.; Zhou, W.; He, Y.; Sun, Z.; Zeng, Q.; Yan, H.; Zheng, Z.; Chen, X.; Tang, Z.; Zhang, J. Design of Efficient Inverted NiOX-Based Three-Terminal Back-Contact All Perovskite Tandem Solar Cells. *Advanced Functional Materials* **2024**, *34* (29), 2401508. https://doi.org/10.1002/adfm.202401508.
(54) Chen, L.-C.; Wu, J.-R.; Tseng, Z.-L.; Chen, C.-C.; Chang, S. H.; Huang, J.-K.; Lee, K.-L.; Cheng, H.-M. Annealing Effect on (FAPbI3)1−x(MAPbBr3)x Perovskite Films in Inverted-Type Perovskite Solar Cells. *Materials* **2016**, *9* (9), 747. https://doi.org/10.3390/ma9090747.
(55) Lin, R.; Wang, Y.; Lu, Q.; Tang, B.; Li, J.; Gao, H.; Gao, Y.; Li, H.; Ding, C.; Wen, J.; Wu, P.; Liu, C.; Zhao, S.; Xiao, K.; Liu, Z.; Ma, C.; Deng, Y.; Li, L.; Fan, F.; Tan, H. All-Perovskite Tandem Solar Cells with 3D/3D Bilayer Perovskite Heterojunction. *Nature* **2023**, *620* (7976), 994–1000. https://doi.org/10.1038/s41586-023-06278-z.
(56) Ibrahim; Shoukat, A.; Aslam, F.; Israr Ur Rehman, M. Emerging Trends in Low Band Gap Perovskite Solar Cells: Materials, Device Architectures, and Performance Optimization. *Molecular Physics* **2024**, *122* (17), e2316273. https://doi.org/10.1080/00268976.2024.2316273.
(57) Mohamadkhani, F.; Javadpour, S.; Taghavinia, N. Improvement of Planar Perovskite Solar Cells by Using Solution Processed SnO2/CdS as Electron Transport Layer. *Solar Energy* **2019**, *191*, 647–653. https://doi.org/10.1016/j.solener.2019.08.067.
